# Possible effects of low testosterone levels on olfactory function in males

**DOI:** 10.1016/j.bjorl.2020.03.001

**Published:** 2020-04-11

**Authors:** Tolga Kırgezen, Uğur Yücetaş, Ela Araz Server, Okan Övünç, Özgür Yiğit

**Affiliations:** a University of Health Sciences, Istanbul Training and Research Hospital, Department of Otorhinolaryngology/Head and Neck Surgery, Istanbul, Turkey; b University of Health Sciences, Istanbul Training and Research Hospital, Department of Urology, Istanbul, Turkey

**Keywords:** Olfactory function, Olfactory dysfunction, Testosterone, Hyposmia, Olfaction

## Abstract

**Introduction:**

Functions attributed to androgens have increased, ranging from the role in hypothalamic–pituitary–gonadal axis and reproductive behaviors to modulation of cognition, mood and some other functions. Sex differences and changes in circulating sex hormones affect human sensory function. In the literature, authors reported this kind of influence for olfaction predominantly in females.

**Objective:**

To investigate the effects of low testosterone levels on olfactory functions in males, in this prospective clinical study.

**Methods:**

Male patients diagnosed with prostate cancer were included. Thirty-nine patients with prostate cancer whose testosterone levels were lower than 50 ng/dL due to castration, were the study group. Thirty-one patients with prostate cancer who were not castrated with testosterone levels higher than 50 ng/dL were selected as the control group. Acoustic rhinometry and peak nasal inspiratory flow tests were performed for all participants; and for evaluation of olfactory function, both groups completed the Connecticut chemosensory clinical research center olfactory test.

**Results:**

The mean ages of the patients and controls were 69.6 ± 7.2 (57–89) and 66.3 ± 5.8 (50–78) years, respectively (*p* = 0.039). There was a significant difference between groups in terms of testosterone levels (*p* < 0.0001). The multivariate logistic regression revealed testosterone level as the only predictive factor determining the difference between the groups. In terms of olfactory parameters, all scores were lower in the emasculated group (butanol threshold test *p* = 0.019, identification *p* = 0.059, and Connecticut center score *p* = 0.029) There was a significant correlation between testosterone levels and olfactory parameters (*p* = 0.023; *p* = 0.025 for identification and Connecticut center scores, respectively).

**Conclusion:**

Low testosterone levels in males have negative effects on olfactory functions. Further molecular research is required to understand the connection between testosterone and olfaction.

## Introduction

The sense of smell is a sensorineural system playing important roles in human life. Olfactory disorders may negatively affect the quality of life, safety and nutrition. Any disease affecting the regions located in the anatomical pathway of the olfactory system from the nasal cavity to the central nervous system (CNS) may be involved in the etiology of olfactory disorders. In addition to these anatomical pathologies, some endocrinological disorders, such as Addison's disease, Turner syndrome or hypothyroidism may cause these disorders.^1^

Sex differences and changes in one's menstrual cycle affect human sensory function even though they do not cause olfactory disorders. Association between human olfactory function and reproductive hormones is complex, and a simple relationship between levels of circulating gonadal hormones and olfaction is rarely present.^2^

Some studies demonstrate that olfactory functions are altered by central and peripheral mechanisms that correlate with changes in steroid hormone levels in females.^2^, ^3^, ^4^, ^5^ Nevertheless, the exact mechanism modulating olfactory perception is unknown. With regard to testosterone and its association with olfaction, we encounter fewer relevant studies in the literature, almost all of which focus on animals.

Functions attributed to androgens have increased in recent years, ranging from playing a well-known role in the hypothalamic–pituitary–gonadal axis and reproductive behaviors to modulation of functions like cognition and mood. Testosterone effects in the nervous system are mediated by androgen or estrogen receptors (after neural aromatization).^6^, ^7^

Hypogonadism is characterized by low levels of testosterone that cause impotence, libido loss, vasoactive symptoms, dyslipidemia, and weight gain; as such, decreased testosterone levels affect many organ systems.^8^

Prostate cancer (PCa) is one of the most common cancers in men. In 1941, Huggins reported the proliferation and growth of the prostate epithelium; since that time androgen deprivation therapy (ADT) has asserted its position as the main management option for advanced PCa.^9^, ^10^

ADT improves the survival rate and quality of life of these patients. Gonadotropin-releasing hormone (GnRH) agonists and antiandrogens are the mostly utilized initial hormonal therapy. ADT causes hypogonadism in PCa patients.^11^

Sex differences and changes in circulating sex hormones affect human sensory function. In the medical literature, authors reported this kind of influence for audition, vision, gustation and olfaction. To the best of our knowledge, we are the first to demonstrate the relationship between testosterone levels and olfaction in human males.

In this study, we intend to demonstrate how low testosterone levels caused by ADT affect olfactory function alongside some nasal parameters.

## Methods

Ethics committee approval was granted from our institutional review board (date: 7th December, 2018, approval number: 1556). After all the participants who were included in the study were informed about the nasal function tests, they provided their informed consent to participate. This prospective and controlled study was conducted between June 2018 and February 2019. We involved male patients diagnosed with PCa who were divided into two groups. Among our sample, 39 patients with PCa whose testosterone levels were lower than 50 ng/dL due to surgical or medical castration performed in our hospital's Urology Clinic constituted the study group, while 31 non-emasculated patients with PCa whose testosterone levels were higher than 50 ng/dL were categorized as the control group.

All possible participants were examined with nasal endoscopy and individuals with a sinonasal pathology that could alter olfactory sensation such as septal deviation, nasal polyposis or acute sinusitis, were excluded from the study. Patients who had undergone nasal surgery or who possessed a history of head trauma and upper respiratory tract infection during examination were excluded.

To obtain objective measures, acoustic rhinometry (AR) (RhinoScan, Manual v. 2.6 ed. 1.1, RhinoMetrics®, Denmark) and peak nasal inspiratory flow (PNIF) tests were performed before and after the use of decongestant nasal spray (0.01% xylometazoline hydrochloride). For evaluation of olfactory function, all participants completed Connecticut chemosensory clinical research center (CCCRC) olfactory test.

Subjects’ age, testosterone level, body mass index, smoking history, accompanying diseases, minimum cross-sectional area (MCA) 1 and 2 values in AR, and PNIF, butanol threshold, odor identification and CCCRC scores were compared between two groups.

### Evaluation of the olfactory function

For the butanol threshold test, diluted butanol solutions were prepared and aligned from 0 to 9, where 0 represented the most concentrated solution and 9 represented the most diluted solution. The strongest concentrate (bottle 0) was 4% butanol in a distilled water solution. Each subsequent dilution (bottles 1–9) was a 1:3 ratio of butanol to distilled water. Patients were presented with two bottles that were identical in appearance, although one contained distilled water and one contained the diluted butanol concentrate. The subjects were instructed to occlude one nostril and place the tip of the first bottle 3 cm beneath the other nostril. Once each subject correctly identified the same butanol concentration five times consecutively, the score was recorded for that nostril. The other nostril was then tested separately, and the scores for both nostrils were averaged to achieve the final score. The possible scores ranged from 0 to 9 points, but all scores of 7 points or higher were recorded as 7 per the CCCRC test.

As an identification test, odors of the CCCRC test that Veyseller et al.^12^ deemed appropriate for use among the Turkish population, were used. Vicks, soap, peanut butter, mothballs, cocoa, coffee, cinnamon, and baby powder were presented to the subjects in opaque bottles. They were then administered a multiple-choice list containing the following distractor items: burnt paper, Vicks, cocoa, baby powder, peanut butter, coffee, mint, cinnamon, soap, mothballs, jam, ketchup, pepper, and plastic. One's ability to sense Vicks indicated an intact trigeminal nerve function; this item was easily identified by all subjects and was not included in the final score. Scores for both nostrils were averaged, and all scores ranging from 0 to 7 were noted.

The butanol threshold and identification scores were averaged for a final composite CCCRC score to determine each subject's olfactory capacity.

### Statistical analysis

The findings were statistically analyzed with the Statistical Package for the Social Sciences, version 18. Each variable's normal distribution suitability was analyzed via visual (histogram and probability graphs) and analytical methods (Kolmogorov-Smirnov and Shapiro–Wilk tests). Descriptive statistics were given as the mean and standard deviation for normally distributed numerical variables and as the median and interquartile range for abnormally distributed numerical variables. An independent *t*-test was implemented to compare two independent variables, while the Mann–Whitney *U* test was applied for variables that did not present a normal distribution. Correlation coefficients and statistical significance for relationships between abnormally distributed numerical variables were measured by the Spearman correlation test, where categorical variables were expressed in numbers and percentages. The differences between the groups were compared using a Chi-Square test and Fisher's exact test. A multivariable logistic regression analysis was performed to determine each variable's independent effect on the outcome variable. The statistical significance level was considered to be less than 0.05.

## Results

A total of 39 patients in Group 1 with low testosterone level and 31 non-emasculated patients in Group 2 (control group) were analyzed, among whom all were diagnosed with PCa. Among the control group, 19 patients received radical prostatectomy, 4 patients received radiotherapy and 8 patients underwent active surveillance. In low testosterone group, 37 patients were emasculated medically (As Luteinizing Hormone Releasing Hormone analogs, 32 patients received leuprolide acetate; 5 patients received goserelin acetate), while 2 patients were emasculated surgically (bilateral orchiectomy).

The mean ages of the patients in Group 1 and Group 2 were 69.6 ± 7.2 (57–89) and 66.3 ± 5.8 (50–78) years, respectively (*p* = 0.039). A significant difference was identified between groups in terms of testosterone levels (*p* < 0.0001), although no statistically significant difference was detected between two groups in terms of other demographic features (Table 1). The multivariate logistic regression revealed testosterone level as the only predictive factor determining the difference between the groups.

**Table 1:** Comparison of demographic characteristics of volunteers.

	Castrated Group (*n* = 39)	Control Group (*n* = 31)	*p*
*Age (year)*			0.039^a^
Mean ± SD	69.6 ± 7.2	66.3 ± 5.8	
Median (IQR), min–max	69 (11), 57–89	67(7), 50–78	

*BMI*			0.772^b^
Mean ± SD	27.2 ± 3.7	27.2 ± 5.0	
Median (IQR), min–max	27(5), 19–37	27(6), 19–41	

*Smoking history*	29 (74.4%)	19 (61.3%)	0.242^c^
*DM*	4 (10.3%)	2 (6.5%)	0.572^c^
*HT*	11 (28.2%)	9 (29%)	0.939^c^
*CAD*	6 (15.4%)	2 (6.5%)	0.243^c^
*COPD*	2 (5.1%)	1 (3.2%)	0.587^d^
*History of malignancy*	5 (12.8%)	1 (3.2%)	0.154^c^

*Testosterone level (ng/dL)*			<0.0001^b^
Mean ± SD	17.7 ± 13.6	370.5 ± 106.6	
Median (IQR), min–max	14 (17), 1–55	370 (139), 124–642	

IQR, interquartile range; BMI, body mass index; DM, diabetes mellitus; HT, hypertension; CAD, coronary artery disease; COPD, chronic obstructive pulmonary disease.

a Independent *t* test.

b Mann–Whitney *U* test.

c Chi-Square test.

d Fisher exact test.

**Figure 1:**
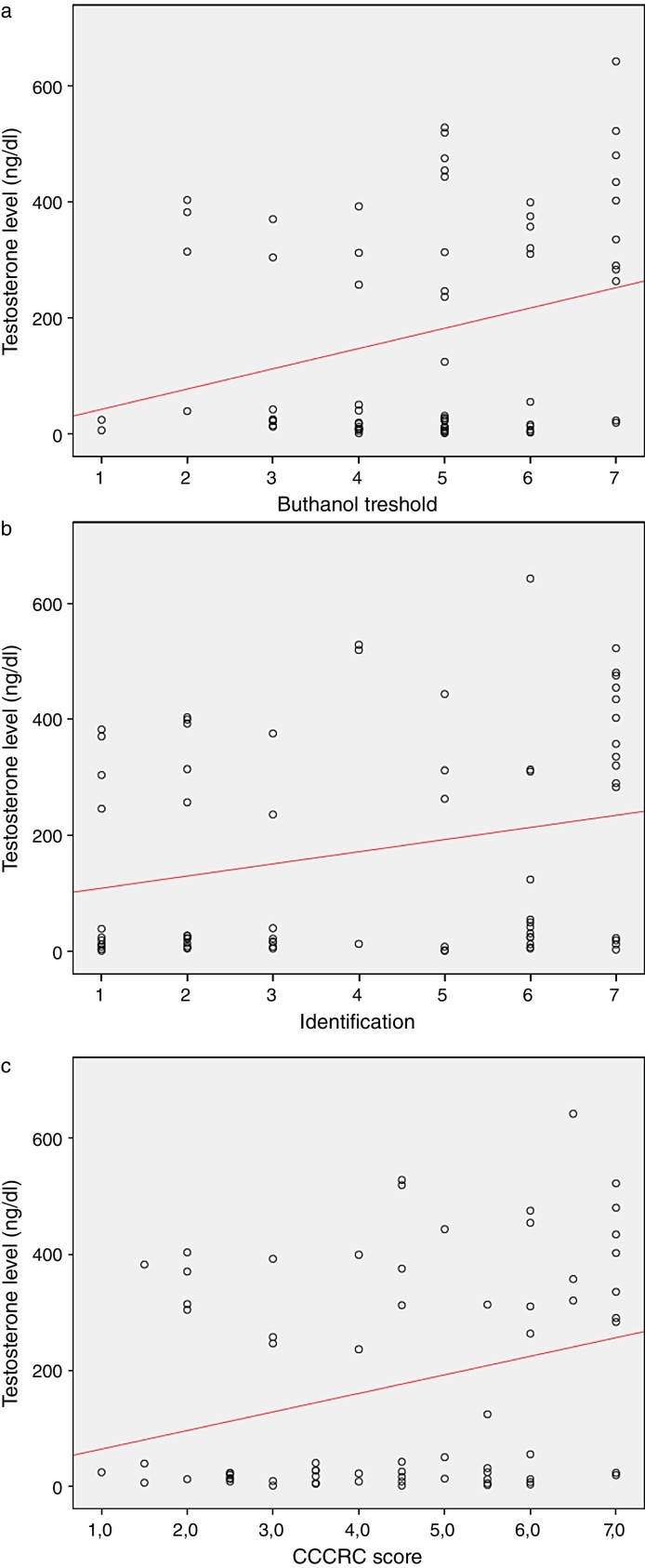
(A) Testosterone level and butanol threshold; (B) testosterone level and odorant identification; (C) testosterone level and CCCRC score.

**Figure 2:**
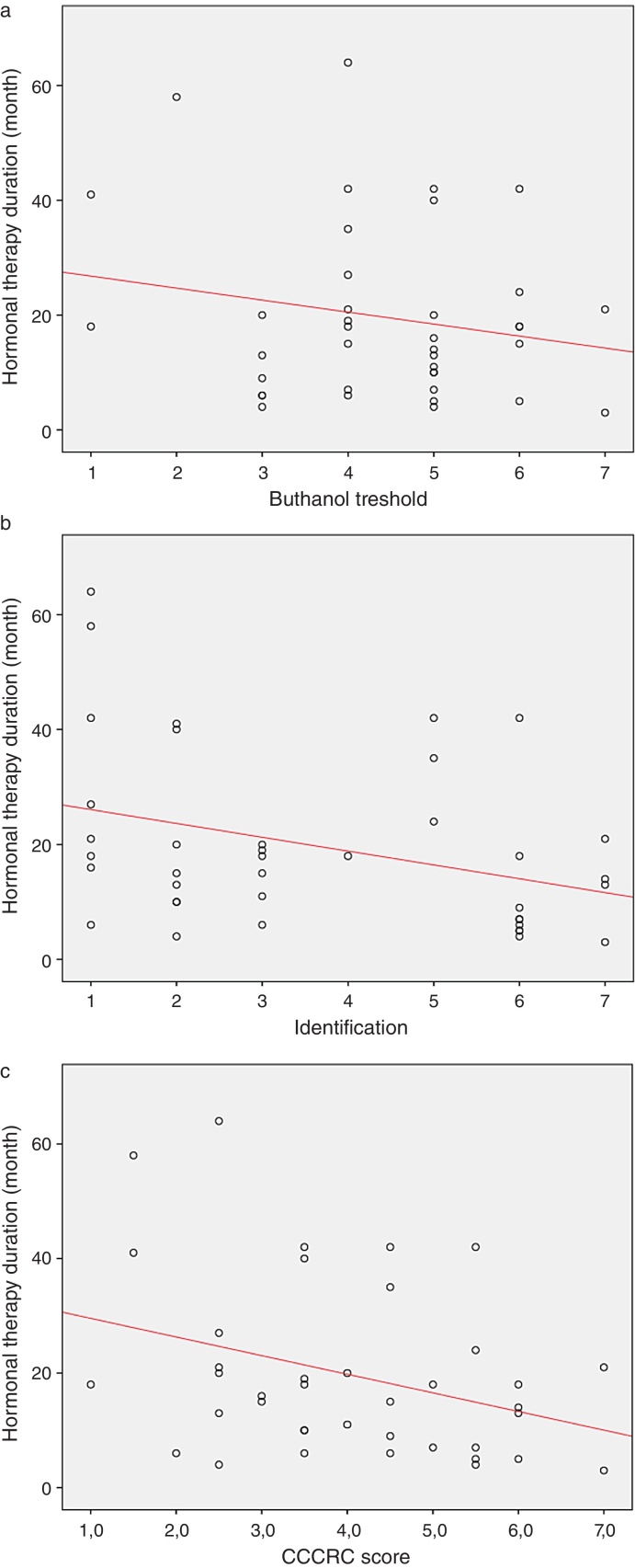
(A) Hormonal therapy duration and butanol threshold; (B) hormonal therapy duration and odorant identification; (C) hormonal therapy duration and CCCRC score.

When both groups were compared in terms of olfactory parameters, all scores were lower in the emasculated group (butanol threshold test *p* = 0.019, identification test *p* = 0.059, and CCCRC score *p* = 0.029) (Table 2). A significant correlation was identified between testosterone level and olfactory parameters (for identification test: *p* = 0.023 and for CCCRC score: *p* = 0.025) (Fig. 1A–C). Further, in the low-testosterone group, a significantly negative correlation was exclusively determined between the duration of hormonal therapy and olfactory parameters in terms of identification (*p* = 0.017) (Fig. 2A–C). Within the emasculated group, the identification and CCCRC scores were lower among the patients whose hormonal therapy duration was longer than 9 months (Table 3).

**Table 2:** Comparison of groups from the point of olfaction.

Castrated group (*n* = 39)	Control Group (*n* = 31)	*p*
Mean ± SD	Mean ± SD	
Median (IQR), min–max	Median (IQR), min–max	
*Butanol threshold*		
4.41 ± 1.41	5.23 ± 1.61	0.019^a^
5 (1), 1–7	5 (3), 2–7	

*Identification*		
3.67 ± 2.18	4.65 ± 2.33	0.059^a^
3 (4), 1–7	5 (5), 1–7	

*CCCRC score*		
4.04 ± 1.55	4.94 ± 1.85	0.029^a^
4 (3), 1–7	5.5 (3.5), 1.5–7	

CCCRC score, Connecticut Chemosensory Clinical Research Center olfactory test; IQR, interquartile range.

a Mann–Whitney *U* test.

**Table 3:** Relationship between hormonal therapy duration and olfactory parameters.

	HT duration ≤9 months (*n* = 11)	HT duration >9 months (*n* = 28)	*p*
*Butanol threshold*			0.648^a^
≤4	6 (54.5%)	13 (46.4%)	
>4	5 (45.5%)	15 (53.6%)	

*Identification*			0.012^a^
≤4	3 (27.3%)	20 (71.4%)	
>4	8 (72.7%)	8 (28.6%)	

*CCCRC score*			0.037^a^
≤4	3 (27.3%)	18 (64.3%)	
>4	8 (72.7%)	10 (35.7%)	

HT, hormonal therapy.

a Chi-Square test.

## Discussion

In our study we found that olfactory function is worse in patients with PCa whose testosterone levels ere lower than 50 ng/dL than in patients with PCa who have testosterone levels higher than 50 ng/dL. This finding implies that testosterone may have an influence on olfactory function, although the question as to how testosterone and olfaction are connected may arise.

Olfactory loss can result from a conductive pathology that prevents inspired odorants from reaching the nose's olfactory cleft or a sensorineural pathology in Olfactory Receptor (OR) neurons or their central projections.

In the literature, we observe hypogonadism with olfactory dysfunction. In Kallmann Syndrome (KS) – otherwise known as congenital hypogonadotropic hypogonadism – the hypogonadism is associated with anosmia.^13^

In KS, gene mutations cause GnRH neurons’ migration failure from the olfactory placode to their hypothalamic destination and olfactory lobe, and olfactory neuron disorder.^14^ The GnRH and olfactory neurons have shared developmental embryologic origins,^15^ both originate from stem cells within the embryonic olfactory placode.^16^ GnRH neurons migrate to the hypothalamus via vomeronasal nerves.^17^, ^18^ Here, we observe the GnRH and olfactory region together.

The classic endocrine feedback loop occurs in the male reproductive system. Hypothalamic GnRH stimulates pituitary Luteinizing Hormone (LH) and Follicle-Stimulating Hormone (FSH), both of which stimulate the testes LH stimulates testosterone secretion by Leydig cells. After castration there may be changes in the feed-back mechanism and neuroendocrine environment. The relation between olfaction and testosterone may be via GnRH in the arc.

Although GnRH and steroid receptors are present in olfactory processing regions across vertebrates. Androgen receptor mRNA levels in the olfactory bulb of cichlidae fishes have been deemed positively correlated with circulating androgen levels.^19^

Neuroendocrine cells that contains GnRH have different origins and functions in nervous system. In the adult brain, these cells are also dispensed in olfactory nerve/bulb and medial olfactory nuclei.^20^ The GnRH cells are also found in ectopic locations, such as the nasal submucosa and connective tissue between the olfactory epithelium (OE) and the olfactory bulb in mice.^21^

The Nervus Terminalis (NT) is an organized GnRH-containing nerve that extends from the nasal cavity to brain regions mediating chemosensory processing and reproduction.^22^, ^23^ Wirsig-Wieshmann et al. reported the GnRH receptor expression in olfactory mucosa and suggested that GnRH modulates the activity of chemoreception-associated cells, that has the potential to alter olfaction.^24^

Kawai et al. suggested that in goldfishes, the NT plays a critic role mediating the olfactory responsiveness of animals.^25^

Neuhaus et al. demonstrated that prostate-specific G Protein-coupled Steroid Receptor's (PSGR) status in prostate cells as an ectopic component of OR superfamily implies functionality because it can be activated by ligands. PSGR transcripts can also be distinguished in human OE.^26^, ^27^

Relations are very complex between neuroendocrine factors and the human olfactory system. The possible connection might not be simple as a direct relation between olfaction and synchronously circulating gonadal hormone levels; rather many possible mechanisms might exist. If this association is not via nasal patency, airflow, or alterations of the olfactory mucu,; then such possibilities may include an influence on nonspecific brain arousal systems, such as the reticular activating system, direct effect on olfactory transduction pathways of CNS, or an indirect influence of other endocrine systems on the olfactory pathways of the CNS.^2^, ^28^

Another possible connection may be a direct effect of the testosterone hormone on olfaction. Sawa Horie et al., suggested de novo synthesis and/or metabolism of sex steroids in olfactory mucosal cells in rats.^29^ Gonadal hormones pass into the brain, and the studies on labeled steroid hormones suggested that hormone retention in the brain has regional specificity. Estrogens and androgens bind to nuclei in the olfactory bulb,^5^, ^30^, ^31^, ^32^ and aromatase enzyme necessary for the androgen to estrogen conversion, is also expressed in olfactory bulb.^33^, ^34^ Kass et al., suggested that sex steroids somehow modulate the activity of primary sensory neurons in the main OE.^35^ The human brain is sensitive to the actions of gonadal steroids; in some olfactory structures, it contains receptors for androgen and estrogen.^36^, ^37^

Altered olfactory sensitivity that correlates with reproductive steroid hormone level changes in females has been reported.^2^, ^3^, ^4^, ^5^ for mainly their positive effects on olfactory function; however, for testosterone some studies in the literature suggested the occurrence of negative effects. Kanageswaran et al. reported the modulatory effects of progesterone and estradiol on odorant-evoked responses in OR neurons in mice.^38^ Kass et al. suggested that gonadal hormones may facilitate detection of odors and discrimination of similar odorants in females although they reduce such detection in males.^35^ Pietras and Moulton^5^ reported that testosterone in supraphysiologic doses improved odor detection ability in gonadectomized female rats (which testosterone's aromatization to estrogens may be a possible mechanism), but male castration does not influence male rats’ sensitivity to either female estrous urine or ethyl acetate.^2^, ^39^, ^40^

We conducted the present study to more thoroughly assess the possible role that testosterone plays in males, and identified decreased olfactory function (i.e. worsened butanol threshold and worsened identification of odors) in males with low testosterone levels. We further identified a significant correlation between testosterone levels and olfactory parameters.

In the literature, it was indicated that both male and female mice that lack circulating gonadal hormones fail to achieve the Go/No-Go task.^41^, ^42^ Kunkhyen et al.^42^; reported that administration of testosterone propionate to castratedmale mice brings urinary odor discrimination capacity back to the pre-gonadectomy levels; gonadal hormones, but not sex, affect the acquisition and maintenance of a Go/No-Go odor discrimination task. Doty and Ferguson-Segall^40^; indicated that castration influences the male rat's ability to improve its odor detection capability over time.

Another possible connection between the testosterone and the olfactory function may be the cognitive functions mediated by gonadal hormones. Bioavailable testosterone level is associated with cognitive function in elderly men.^43^ Testosterone supplementation improves working memory in elderly men who have reduced free testosterone levels.^44^ In male rats Gibbs et al.^45^; reported that testosterone has significant influences on specific cognitive domains.

We also examined the nasal airway to detect the conductive problems that might be responsible for olfactory dysfunction. The tests conducted herein (AR and PNIF) provided us data to make comparisons between testosterone levels. The MCA 1–2 values in AR and PNIF did not reveal any significant differences between the two groups, which points out a more possible sensorineural mechanism of the olfactory dysfunction.

Nearly all animal and human studies that concern the influence of sex and circulating sex hormones on main olfactory function have assessed odorant detection thresholds. We additionally studied our subjects’ capacity to identify odorants and determined that the low-testosterone group achieved significantly worse results than the non-emasculated group on the butanol threshold test. Odor identification was also worse in the low-testosterone levels. Testosterone level was found to be significantly correlated with one's olfactory identification capacity.

The overall duration with low testosterone levels is important for patients’ development of olfactory dysfunction. We determined a significantly negative correlation that exists between ADT duration and odor identification.

An exact connection mechanism between testosterone hormone and olfactory dysfunction could not be determined in animal and laboratory studies which were published previously. To the best of our knowledge this is the first human study which shows the effects of testosterone on olfaction in human males. This study also revealed some definitive results and related correlations. It does not mean that “no testosterone, no olfaction”, although a connection seems possible.

A study might be designed with a patient group with reduced testosterone levels, whose olfaction tests should be performed before and after replacement of testosterone hormone. But such a design is not possible for these patient groups. Thus, to obtain more definitive results, the olfactory evaluation and comparison must be performed both before and after the therapeutic castration of each individual patient.

## Conclusion

This study revealed that testosterone levels may have effects on olfactory function in males. In males, a low testosterone level negatively affects the olfactory threshold and identification of volatile odorants. Lengthening of the low testosterone duration worsens the odor identification. Further molecular researches are required in order to understand the true and exact mechanism that provides the connection between testosterone levels and olfaction.

## Ethical approval

All procedures performed in studies involving human participants were in accordance with the ethical standards of the institutional research committee (Istanbul Training and Research Hospital's Ethics Committee; reference number: 1556) and with the 1964 Helsinki declaration and its later amendments or comparable ethical standards.

## Informed consent

Informed consent was obtained from all individual participants included in the study.

## Disclosures

The authors declared no potential conflicts of interest with respect to the research, authorship, and publication of this article.

## Funding

The authors received no financial support for the research, authorship, and publication of this article. This manuscript was presented in 15th Turkish Rhinology Congress, 4th–7th April, 2019, in Antalya, Turkey.

## Conflicts of interest

The authors declare no conflicts of interest.
